# Academic-associated emotions before and during the COVID-19-related online semester – a longitudinal investigation of first-year medical students

**DOI:** 10.3205/zma001370

**Published:** 2020-12-03

**Authors:** Sabine Polujanski, Ann-Kathrin Schindler, Thomas Rotthoff

**Affiliations:** 1 Universität Augsburg, Medizinische Fakultät, Lehrstuhl für Medizindidaktik und Ausbildungsforschung, DEMEDA, Augsburg, Germany

**Keywords:** academic-associated emotions, emotion regulation, online semester, COVID-19

## Abstract

**Background: **Due to the COVID-19 pandemic, students have been confronted with an online semester. Because of the special requirements, online teaching can trigger negative emotions, which can have an unfavourable impact on the learning process and which therefore need to be regulated. This study investigates academic-associated emotions and the regulation of those emotions both before (December 2019) and during (June 2020) the online semester for first-year medical students.

**Methods: **Questionnaire data (t1=Dec 2019; t2=Jun 2020) regarding academic-associated emotions and emotion regulation, taken from a longitudinal research project (Experienced Learning Medicine Augsburg; ELMA) at the University of Augsburg, was used. At t2, the students were also asked, as future physicians, to name their three most significant emotions regarding their studies, taking into account the COVID-19 situation.

**Results:** Longitudinal analyses (Wilcoxon tests) showed few changes in academic-associated emotions. The emotions happy (*r*=.32) and proud (*r*=.33) increased significantly with moderate effects at the online semester. There also was an increased, but still low suppression of emotions (*r*=.22) at t2. The future physicians were most often curious, grateful and afraid about their medical studies with regard to the COVID-19 situation. Overall, medical studies were more often associated with positive than negative emotions during the online semester.

**Conclusions: **The results show that the online semester did not have any worrying impacts on academic-associated emotions and emotion regulation. There was even some indication that students might benefit from online teaching formats.

## Zusammenfassung

**Hintergrund:** Infolge der COVID-19 Pandemie wurden Studierende mit einem Online-Semester konfrontiert. Online Lehre kann aufgrund der besonderen Anforderungen negative Emotionen auslösen, die sich ungünstig auf den Lernprozess auswirken können und deshalb entsprechend reguliert werden sollten. Diese Studie untersucht Emotionen gegenüber dem Studium und deren Regulation vor (Dezember 2019) und im Online-Semester (Juni 2020) bei Medizinstudierenden im ersten Studienjahr.

**Methode: **Fragebogendaten (t1=Dez 2019; t2= Jun 2020) zum emotionalen Erleben des Studiums und der Emotionsregulation wurden aus einem longitudinalen Forschungsprojekt (Experienced Learning Medicine Augsburg, ELMA) der Universität Augsburg verwendet. Zusätzlich wurden die Studierenden in ihrer speziellen Situation als zukünftige Ärztinnen und Ärzte zu t2 aufgefordert, die drei bedeutendsten Emotionen gegenüber ihrem Studium unter Berücksichtigung der COVID-19 Situation zu benennen.

**Ergebnisse: **Die längsschnittlichen Analysen (Wilcoxon-Tests) zeigen kaum Veränderungen der emotionalen Wahrnehmung des Studiums. Die Emotionen fröhlich (*r*=.32) und stolz (*r*=.33) nehmen mit mittleren Effekten im Online-Semester signifikant zu. Im Online-Semester zeigt sich eine verstärkte, jedoch geringe Unterdrückung von Emotionen (*r*=.22). Angehende Ärztinnen und Ärzte stehen ihrem Studium mit Blick auf die COVID-19 Situation am häufigsten neugierig, dankbar und besorgt gegenüber. Insgesamt wurde das Studium im Online-Semester häufiger mit positiven, als mit negativen Emotionen assoziiert.

**Schlussfolgerungen: **Die Ergebnisse zeigen, dass das Online-Semester keine bedenklichen Auswirkungen auf das emotionale Erleben des Studiums und die Emotionsregulation hat, sie deuten sogar daraufhin, dass Studierende von Online-Lehr-Formaten profitieren können.

## 1. Background

The COVID-19-related online semester has placed unusual demands on students (e.g. less social integration and higher self-regulation of the learning process than in face-to-face teaching), which could trigger negative emotions (e.g. frustration) [1], [2]. This can be problematic, because a successful learning process, including high academic performance [3] and favourable motivational situation [4], [5], is ideally accompanied by positive emotions. Emotion regulation strategies enable individuals to consciously influence the intensity, duration and quality of the experience and expression of emotions [6], [7]. The strategy suppression involves repressing emotional expression, while cognitive reappraisal is the active cognitive reinterpretation of emotional situations [8]. In the latter, the meaning of an emotionally triggering situation is reinterpreted [6], which should lead to a more positive emotional reaction [8]. For example, frustration caused by the complexity of learning materials might be avoided or mitigated by viewing the situation not as a threat, but as an opportunity to acquire knowledge. Higher performing students have been found to more often apply cognitive reappraisal [9], which simultaneously reduces negative emotions [10].

This study examines academic-associated emotions and their regulation in first-year medical students before and during the COVID-19-related online semester.

## 2. Method

### 2.1. Design

The study used longitudinal data, which had been voluntarily provided via questionnaire, that was collected by the Experienced Learning Medicine Augsburg (ELMA) research project as part of a newly founded model study programme. The questionnaires were ethically approved. The data was collected in December 2019 (t1) and June 2020 (t2). Online teaching (Moodle platform) was already established in the attendance semester (WS2019/20). The design processes for the online learning units and synchronous teaching via Zoom are accompanied by medical didactics.

#### 2.2. Sample

At t1, 71 students provided responses; at t2, there were 75. Longitudinal matching allowed 65 complete datasets to be integrated. For sociodemographic data, see table 1 (Tab. 1).

#### 2.3. Measures

21 representative academic-associated emotions (10 positive, 11 negative) from the Medical Emotion Scale (MES; 5-point Likert scale from 0-not at all to 4-very strong) [11] were questioned.

The emotion regulation strategies *suppression* (4 items, α=.74) and *cognitive reappraisal* (6 items, α=.76) were recorded by the mean values of the Emotion Regulation Questionnaire (ERQ; 7-point Likert scale ranging from 0-not at all to 6-absolutely) [8], [12].

In their role as future physicians, the students were also asked in the June survey to complete the following statement: “The COVID-19 situation makes me feel… about my medical studies. Please select the three most appropriate emotions.”

#### 2.4. Analyses

The data was analysed descriptively and using Wilcoxon tests (ordinal scale level and restricted normal distribution).

## 3. Results

The longitudinal analyses of academic-associated emotions are shown in table 2 (Tab. 2).

The emotions *happy* and *proud* increased with a moderate effect size, and *disappointed* increased with a small effect size. The emotion regulation strategy *suppression* increased significantly (*r*=.22, *Mdn_Dec_*=2.00, *Mdn_June_*=2.75, *z*=-2.56, *p*=.01), but there were no significant changes for *cognitive reappraisal* (*Mdn_Dec_*=3.67, *Mdn_June_*=3.50, *z*=-.66, *p*=.51).

**Figure 1:**
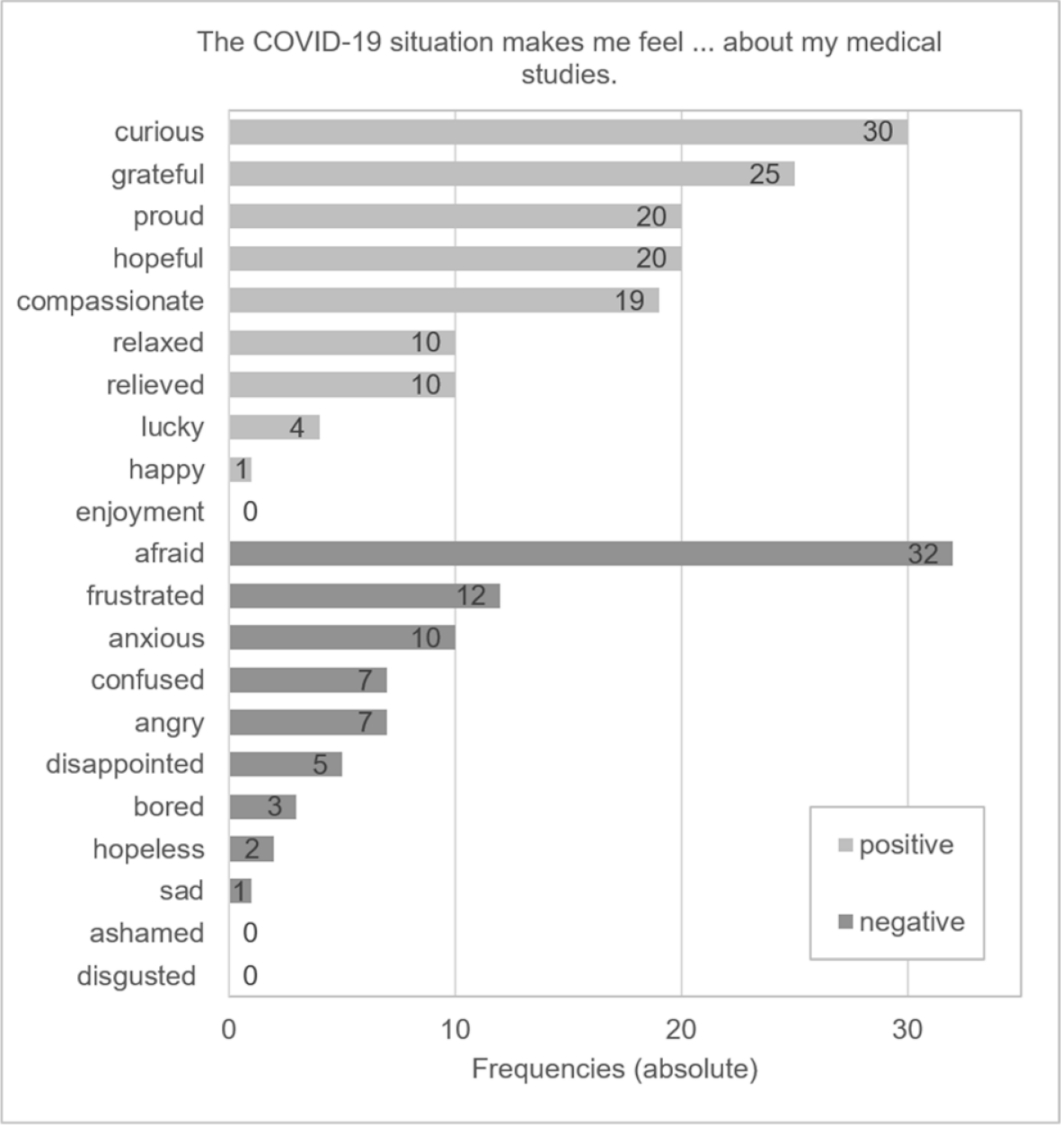
Emotional perception of medical studies in regard to the COVID-19 situation. Notes: Reports were in response to the prompt “The COVID-19 situation makes me feel … about my medical studies. Please select the three most appropriate emotions.” Date of measurement=June 2020; *n*=75.

*Curiosity, gratitude* and *being afraid* were the emotions most frequently described by the future physicians in regard to the COVID-19 situation. Overall, despite the COVID-19 situation, medical studies were more often associated with positive than negative emotions (see figure 1 (Fig. 1)).

## 4. Discussion

While most academic-associated emotions did not change longitudinally, *happy* and *proud* did increase significantly during the COVID-19-related online semester. This might be because of the free allocation of time, individual adaptation to the pace of learning and the time saved by not commuting [13], [14]. This latter reason was mentioned as a form of relief in an additional scale in the questionnaire that assessed burdens and reliefs. Semester-related teaching evaluation results across all courses also showed a high level of satisfaction with online teaching. The increase in pride could be related to the completion of a systemically relevant training, although there is no explanatory data available to support this hypothesis.

*Disappointment* also increased significantly during the online semester, but with only a small effect size. This could be due to the elimination of practical teaching units and reduced peer contact, both were reported as burdens. Presumably, the COVID-19 online semester led to a severely different student life, which might also be a reason for increased disappointment.

The emotion regulation strategy *suppression* saw a significant increase, but with a small effect size, which might be related to the home learning environment during the online semester, which was perceived as a burden.

When asked which three emotions towards medical studies were most triggered by the COVID-19 situation, *curiosity, gratitude* and *being afraid* were most frequently mentioned. Curiosity might refer to the many unknown variables associated with the pandemic [15] and the possibility of acquiring new knowledge [16], while gratitude might relate to the benefits of the online semester described above [13], [14]. However, examinations without prior class attendance were seen by a majority of students as a burden and thus possibly an indicator of being afraid*,* but the data does not allow for a causal explanation and so these hypotheses should be treated as a cautious interpretation.

The results suggest that the exclusive online teaching – at least, after a short period of time – had no worrying impact on the students’ emotions. However, it should be noted that the data is from a model study programme currently being developed with a small student cohort.

## 5. Future research

With regard to the ongoing significant reduction in face-to-face teaching, the student cohort will continue to be monitored longitudinally, with a view to a (gradual) reintegration process [17]. There will also be additional investigation of study–life balance to better clarify emotional developments.

## Competing interests

The authors declare that they have no competing interests. 

## Figures and Tables

**Table 1:**
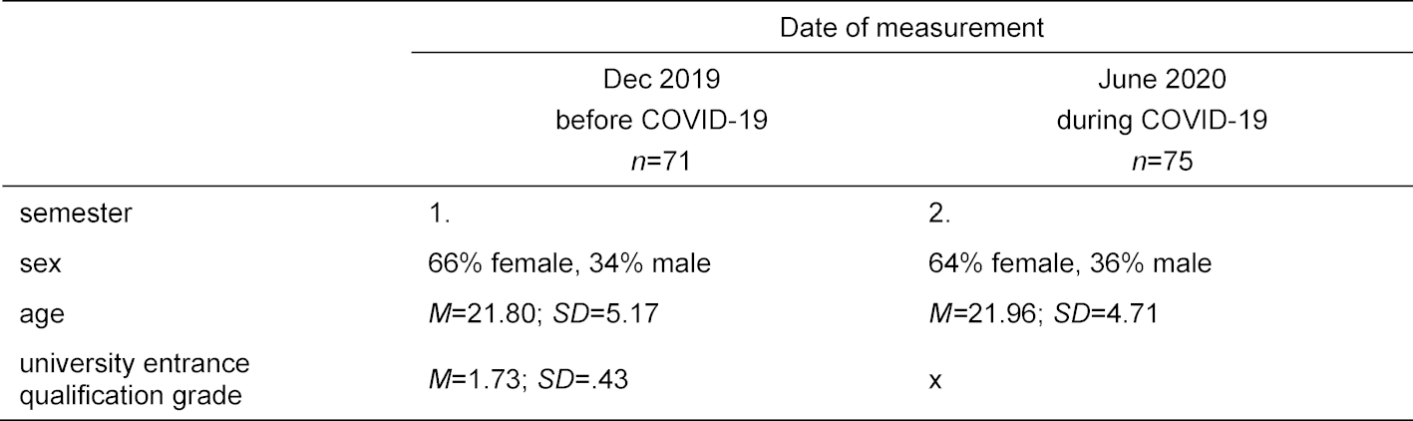
Sociodemographic statistics

**Table 2:**
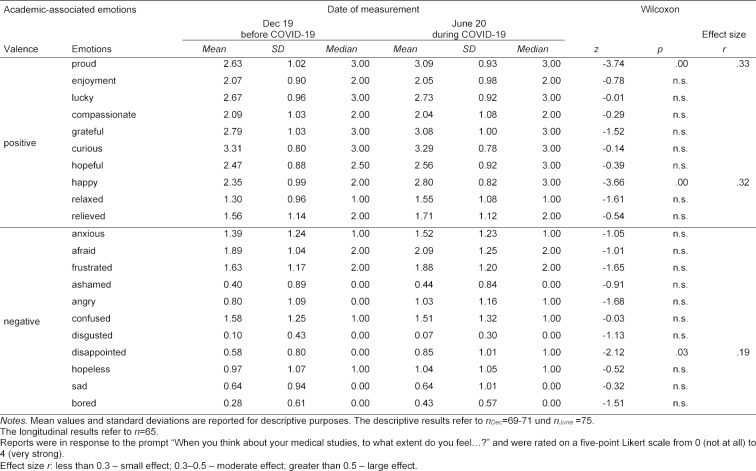
Longitudinal development of academic-associated emotions before and during the COVID-19-related online semester
